# Supplementary material for: Sexually Dimorphic Response to Dietary Restriction‐Induced Longevity and Muscle Preservation in Nothobranchius furzeri
Source: Aging Cell. 2026 Nov 5;25(11):e70791. doi: 10.1111/acel.70791 (PMC13740022; doi:10.1111/acel.70791)
Supplement: Supplementary file 1 [file ACEL-25-e70791-s007.pdf]

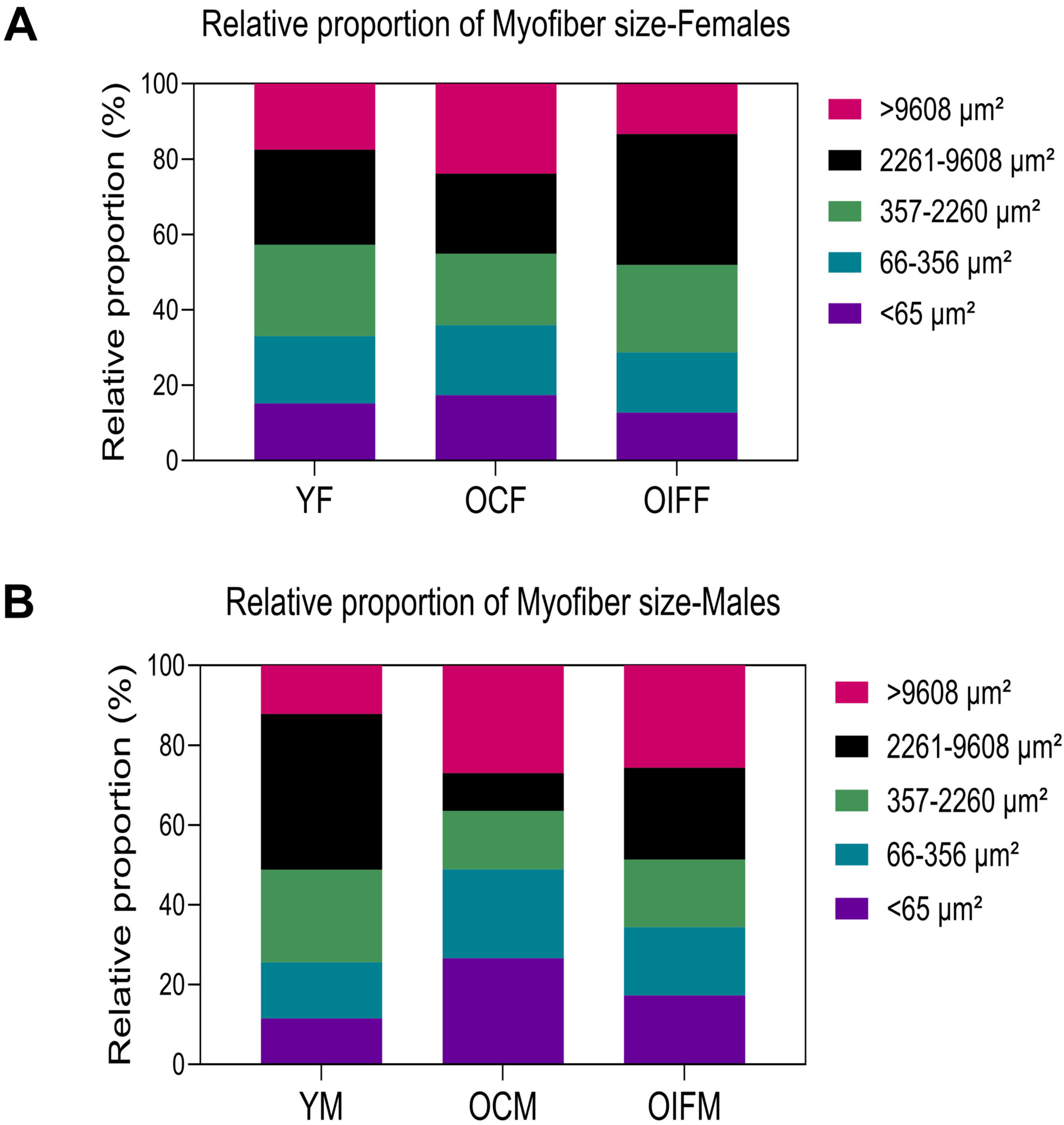
A
Relative proportion of Myofiber size-Females
Relative proportion (%)
100
80
60
40
20
0
YF
OCF
OIFF
>9608 μm²
2261-9608 μm²
357-2260 μm²
66-356 μm²
<65 μm²
B
Relative proportion of Myofiber size-Males
Relative proportion (%)
100
80
60
40
20
0
YM
OCM
OIFM
>9608 μm²
2261-9608 μm²
357-2260 μm²
66-356 μm²
<65 μm²

Fig. S1

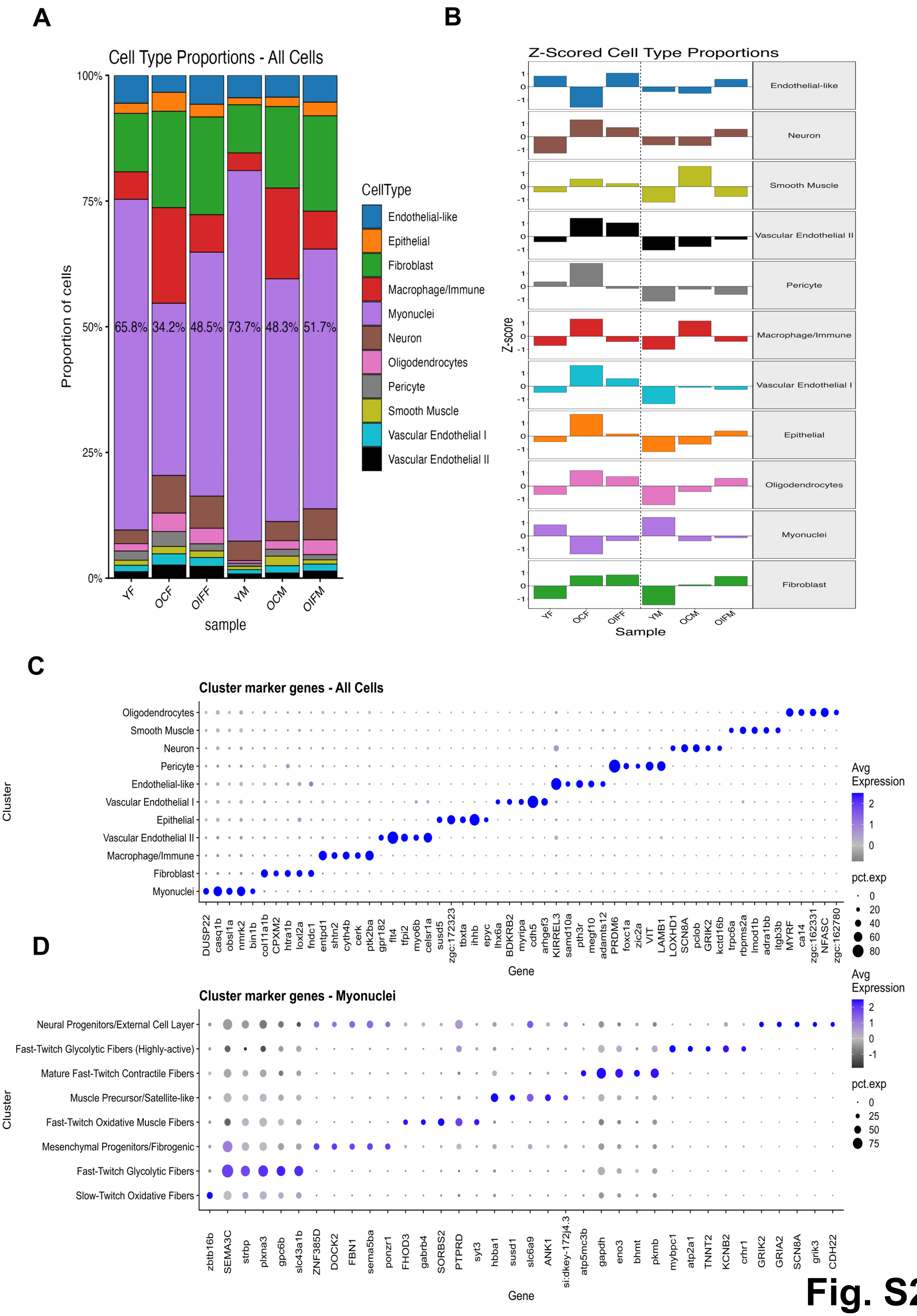
A
B
Cell Type Proportions - All Cells
100%
75%
50%
25%
0%
Proportion of cells
65.8%
34.2%
48.5%
73.7%
48.3%
51.7%
YF
OCF
OIFF
YM
OCM
OIFM
sample
CellType
Endothelial-like
Epithelial
Fibroblast
Macrophage/Immune
Myonuclei
Neuron
Oligodendrocytes
Pericyte
Smooth Muscle
Vascular Endothelial I
Vascular Endothelial II
Z-Scored Cell Type Proportions
Z-score
Endothelial-like
Neuron
Smooth Muscle
Vascular Endothelial II
Pericyte
Macrophage/Immune
Vascular Endothelial I
Epithelial
Oligodendrocytes
Myonuclei
Fibroblast
Sample
C
Cluster marker genes - All Cells
Cluster
Oligodendrocytes
Smooth Muscle
Neuron
Pericyte
Endothelial-like
Vascular Endothelial I
Epithelial
Vascular Endothelial II
Macrophage/Immune
Fibroblast
Myonuclei
DUSP22 casq1b obsl1a nmrk2 bin1b col11a1b CPXM2 htra1b loxl2a fndc1 entpd1 shtn2 cyth4b cerk ptk2ba gpr182 flt4 tfpi2 myo6b celsr1a susd5 zgc:172323 tbxta ihhb epyc lhx6a BDKRB2 myripa cdh5 arhgef3 KIRREL3 samd10a pth3r megf10 adamts12 PRDM6 foxc1a zic2a VIT LAMB1 LOXHD1 SCN8A pclob GRIK2 kctd16b trpc6a rbpms2a lmod1b adra1bb itgb3b MYRF ca14 zgc:162331 NFASC zgc:162780
Gene
Avg Expression
2
1
0
pct.exp
0
20
40
60
80
D
Cluster marker genes - Myonuclei
Cluster
Neural Progenitors/External Cell Layer
Fast-Twitch Glycolytic Fibers (Highly-active)
Mature Fast-Twitch Contractile Fibers
Muscle Precursor/Satellite-like
Fast-Twitch Oxidative Muscle Fibers
Mesenchymal Progenitors/Fibrogenic
Fast-Twitch Glycolytic Fibers
Slow-Twitch Oxidative Fibers
zbtb16b SEMA3C strbp plxna3 gpc6b slc43a1b ZNF385D DOCK2 FBN1 sema5ba ponzr1 FHOD3 gabrb4 SORBS2 PTPRD syt3 hbba1 susd1 slc6a9 ANK1 si:dkey-172j4.3 atp5mc3b gapdh eno3 bhmt pkmb mybpc1 atp2a1 TNNT2 KCNB2 crhr1 GRIK2 GRIA2 SCN8A grik3 CDH22
Gene
Avg Expression
2
1
0
-1
pct.exp
0
25
50
75

Fig. S2

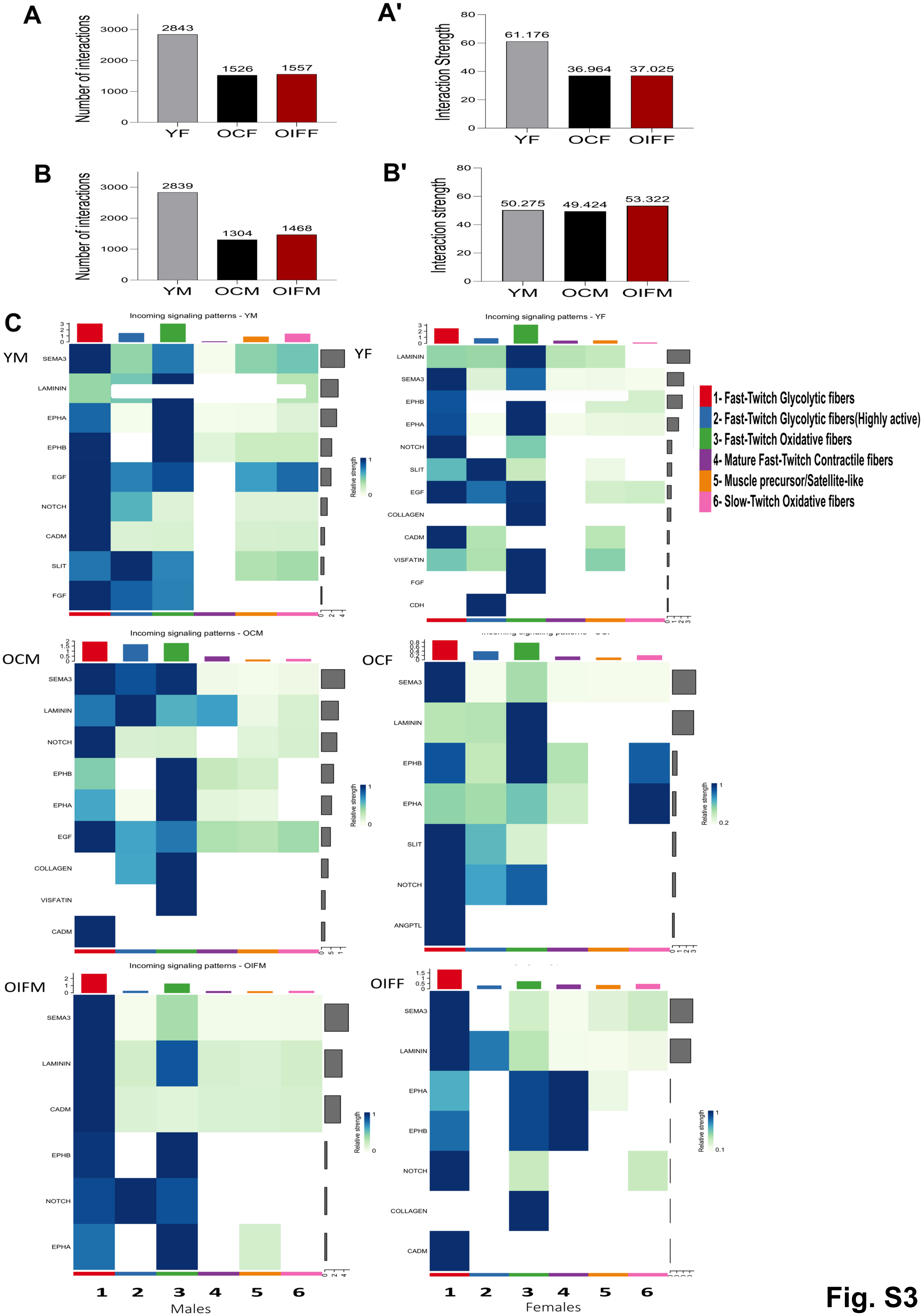

A
Number of interactions
2843
1526
1557
YF
OCF
OIFF
A'
Interaction Strength
61.176
36.964
37.025
YF
OCF
OIFF
B
Number of interactions
2839
1304
1468
YM
OCM
OIFM
B'
Interaction strength
50.275
49.424
53.322
YM
OCM
OIFM
C
Incoming signaling patterns - YM
YM
SEMA3
LAMININ
EPHA
EPHB
EGF
NOTCH
CADM
SLIT
FGF
Relative strength
Incoming signaling patterns - YF
YF
LAMININ
SEMA3
EPHB
EPHA
NOTCH
SLIT
EGF
COLLAGEN
CADM
VISFATIN
FGF
CDH
1- Fast-Twitch Glycolytic fibers
2- Fast-Twitch Glycolytic fibers(Highly active)
3- Fast-Twitch Oxidative fibers
4- Mature Fast-Twitch Contractile fibers
5- Muscle precursor/Satellite-like
6- Slow-Twitch Oxidative fibers
Incoming signaling patterns - OCM
OCM
SEMA3
LAMININ
NOTCH
EPHB
EPHA
EGF
COLLAGEN
VISFATIN
CADM
OCF
SEMA3
LAMININ
EPHB
EPHA
SLIT
NOTCH
ANGPTL
Incoming signaling patterns - OIFM
OIFM
SEMA3
LAMININ
CADM
EPHB
NOTCH
EPHA
OIFF
SEMA3
LAMININ
EPHA
EPHB
NOTCH
COLLAGEN
CADM
1 2 3 4 5 6
Males
1 2 3 4 5 6
Females

Fig. S3

A- Principal Component Analysis

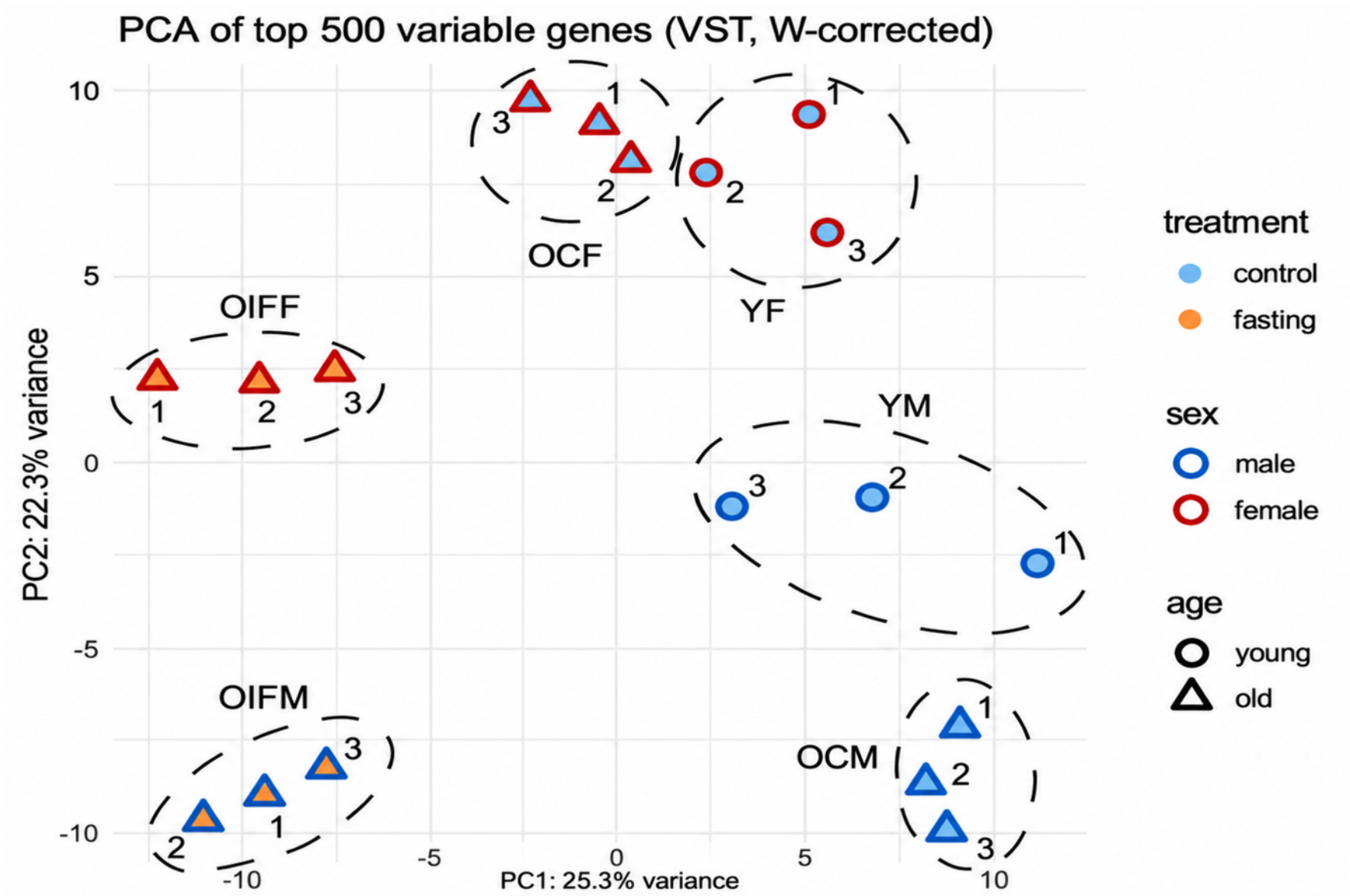

B- Age effect in male

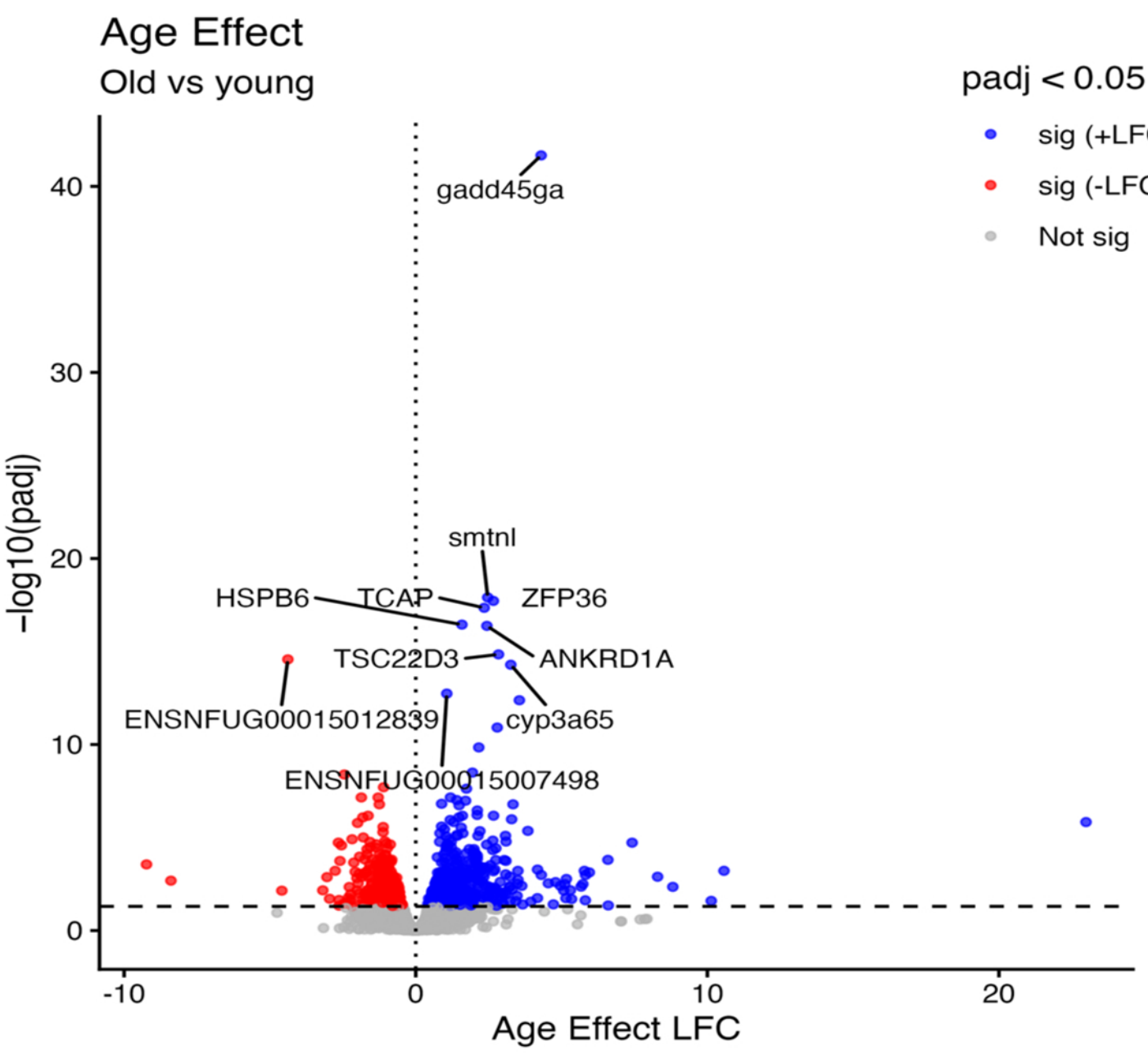

C- Age effect in females

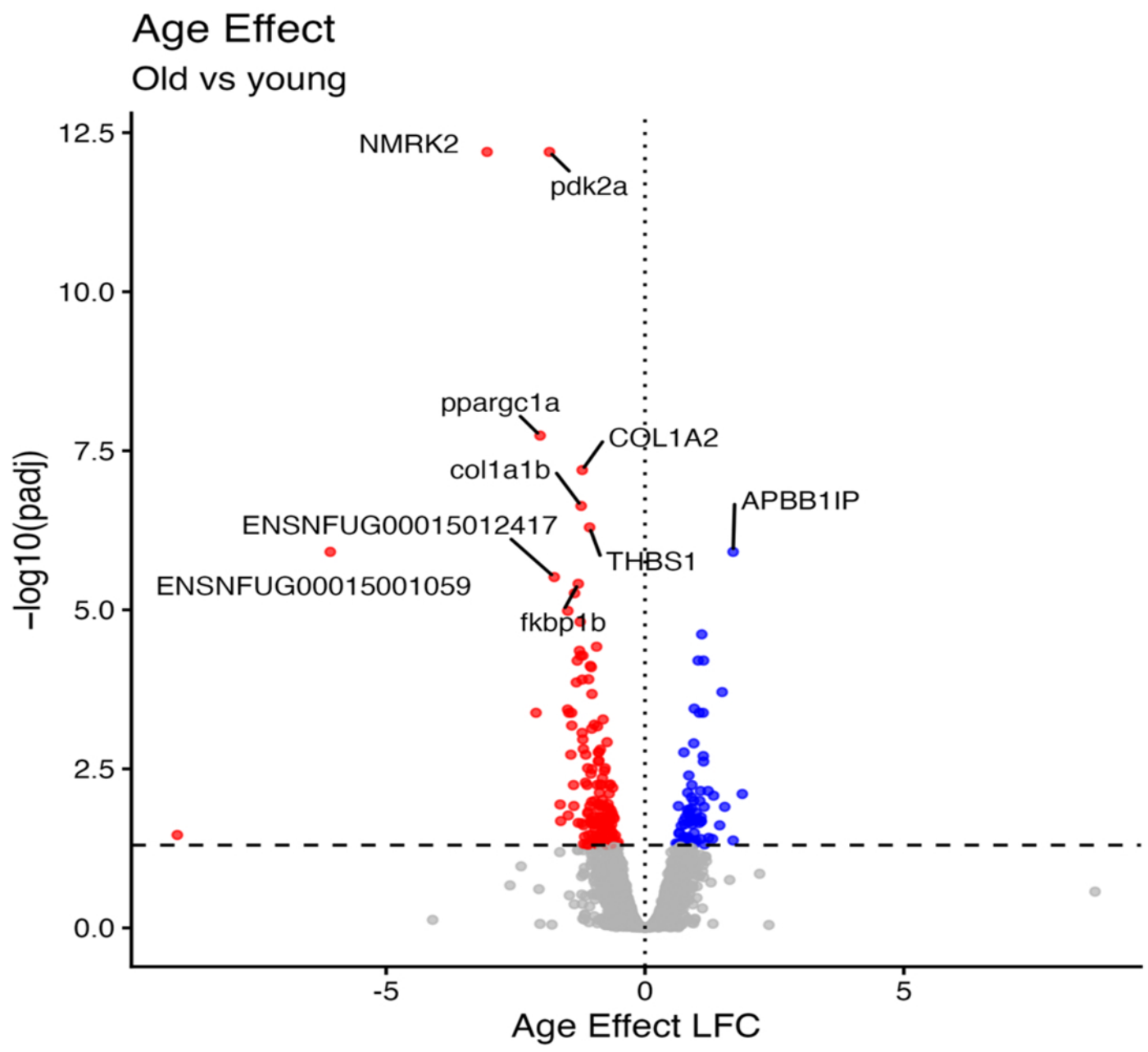

D- IF effect in male

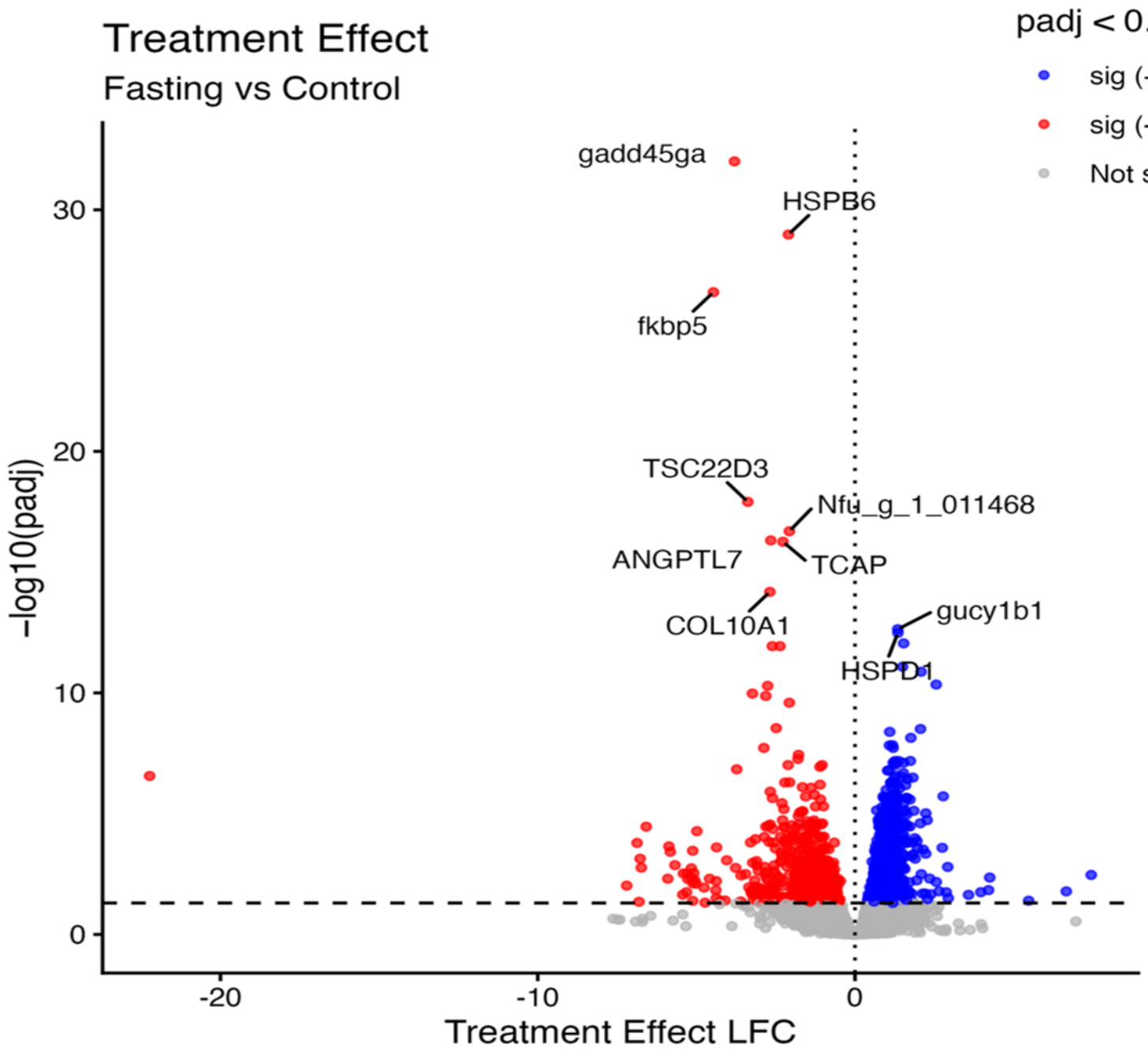

E- iF effect in male

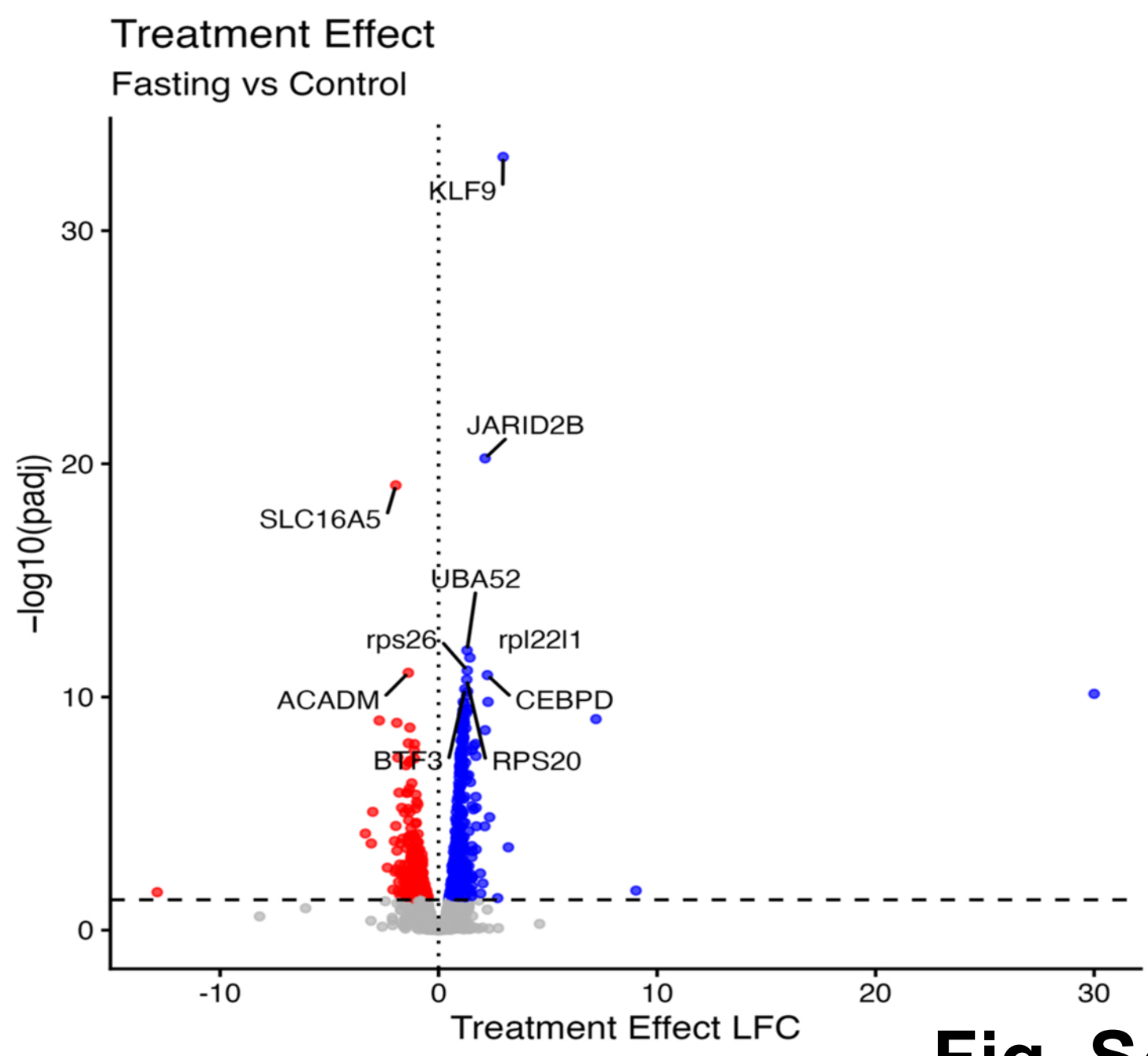

Fig. S4
